# Antenatal care visits and associated factors in Senegal: a multilevel poisson regression analysis of the 2023 DHS survey

**DOI:** 10.3389/fgwh.2025.1524361

**Published:** 2025-05-27

**Authors:** Habtu Kifle Negash, Ashebir Mamay Gebiru, Mihret Getnet, Mihret Melese, Berihun Agegn Mengistie, Desalegn Anmut Bitew, Tilahun Nega Godana, Yosef Belay Bizuneh, Amare Belete Getahun, Mikias Mered Tilahun, Nebebe Demis Baykemagn, Worku Chekol Tassew

**Affiliations:** ^1^ Department of Human Anatomy, School of Medicine, College of Medicine and Health Sciences, University of Gondar, Gondar, Ethiopia; ^2^ Department of Epidemiology and Biostatistics, Institute of Public Health, College of Medicine and Health Sciences, University of Gondar, Gondar, Ethiopia; ^3^ Departments of Health Informatics, Teda Health Science College, Gondar, Ethiopia; ^4^ Department of Human Physiology, School of Medicine, College of Medicine and Health Sciences, University of Gondar, Gondar, Ethiopia; ^5^ Department of General Midwifery, School of Midwifery, College of Medicine and Health Sciences, University of Gondar, Gondar, Ethiopia; ^6^ Department of Reproductive Health, College of Medicine and Health Sciences, University of Gondar, Gondar, Ethiopia; ^7^ Department of Internal Medicine, School of Medicine, College of Medicine and Health Sciences, University of Gondar, Gondar, Ethiopia; ^8^ Department of Anesthesia, School of Medicine, College of Medicine and Health Sciences, University of Gondar, Gondar, Ethiopia; ^9^ Department of Optometry, School of Medicine, University of Gondar, Comprehensive Specialized Hospital, Gondar, Ethiopia; ^10^ Department of Health Informatics, Institute of Public Health, College of Medicine and Health Sciences, University of Gondar, Gondar, Ethiopia; ^11^ Department of Medical Nursing, Teda Health Science College, Gondar, Ethiopia

**Keywords:** antenatal care, multilevel poisson regression, Senegal, demographic health survey, 2023

## Abstract

**Background:**

Maternal health is crucial for women's well-being during pregnancy, childbirth, and postpartum. Antenatal care (ANC) is essential for monitoring health and preventing complications, yet only 35.5% of women in low- and middle-income regions attend the recommended ANC visits. This study explores the socioeconomic and regional factors influencing ANC visits in Senegal.

**Methods:**

Data from the 2023 Senegal Demographic and Health Survey (DHS) on maternal health were analyzed. A stratified two-stage sampling design selected 400 enumeration areas, and 4,543 women aged 15–49 participated. Independent variables included age, education, wealth, healthcare access, and region. Multilevel Poisson regression in Stata 17 was used to assess factors affecting ANC visits, considering both individual and community-level variables to examine regional disparities and healthcare access.

**Results:**

Among the 4,543 women, 97.63% attended at least one ANC visit, but only 7.69% completed eight or more. Wealth, media access, education, and regional location significantly influenced ANC attendance. Women from middle-income households attended 6% more visits (AIRR: 1.06, 95% CI: 1.02–1.11), and those from wealthy households attended 8% more (AIRR: 1.08, 95% CI: 1.03–1.14). Women with media access attended 11% more visits (AIRR: 1.11, 95% CI: 1.06–1.15). Regional disparities were evident, with women in Thiès (AIRR: 0.80, 95% CI: 0.73–0.87), Matam (AIRR: 0.83, 95% CI: 0.75–0.92), and Kédougou (AIRR: 0.83, 95% CI: 0.75–0.91) attending fewer visits compared to those in Dakar.

**Conclusions:**

This study identifies significant socioeconomic and regional disparities in ANC utilization in Senegal. Wealth, education, media access, and regional location strongly influence ANC attendance. Targeted policies focusing on maternal health education, healthcare infrastructure, and financial support, particularly in underserved areas, are necessary to improve ANC access and maternal and child health outcomes.

## Introduction

Maternal health is the cornerstone of public health, encompassing women's well-being during pregnancy, childbirth, and postpartum while prioritizing safety for both mother and child (1). A core component of maternal health is antenatal care (ANC), which the World Health Organization (WHO) defines as “care provided by skilled healthcare professionals to pregnant women and adolescent girls to ensure the best health conditions for both mother and baby” (2).

ANC involves routine check-ups to monitor mother and fetus health, allowing for early complication detection and providing crucial health education on pregnancy and parenting (3). Moreover, ANC contacts offer critical guidance on nutrition, vaccinations, screenings, and emotional support while facilitating timely referrals to specialized care when necessary (4).

WHO envisions a world where every pregnant woman and newborn has access to quality care throughout pregnancy, childbirth, and the postnatal period (5). To achieve this, it advocates for high-quality ANC services as part of a broader global goal toward equitable, people-centered universal health coverage (5). Despite these efforts, approximately 15% of pregnancies experience life-threatening complications requiring immediate skilled intervention (6).This statistic is especially troubling in low- and middle-income countries, where complications during pregnancy and childbirth are leading causes of death and disability among reproductive-age women (7).

Multiple studies have demonstrated a positive correlation between ANC visits and improved maternal and neonatal health outcomes. Research indicates that increased ANC visits reduce both maternal and neonatal morbidity (8) and mortality (9–11). For instance, a single ANC visit is associated with a 1.04% reduction in neonatal mortality and a 1.07% reduction in infant mortality. Achieving at least four ANC visits with one provided by a skilled healthcare professional further decreases these risks by an additional 0.56% and 0.42%, respectively. Moreover, a single ANC visit lowers the probability of low birth weight by 3.82%, while additional visits reduce risks of stunting and underweight by 4.11% and 3.26%, respectively (12). While ANC visits improve health outcomes, their effectiveness also depends on the quality of care, including the healthcare provider's skills and the availability of essential services (13).

Unfortunately, only 35.5% of pregnant women in low- and middle-income regions attend ANC at least four times, often due to financial barriers, limited facility access, and lack of awareness. Socioeconomic status, parental education, rural residence, and maternal age also affect ANC attendance, highlighting the urgent need for improved community awareness and strengthened health education programs (14). In many developing countries, maternal mortality persists as a significant public health challenge despite global initiatives. In sub-Saharan Africa, approximately 66% of global maternal deaths occur, with persistently high maternal mortality ratios despite national health insurance and safe motherhood efforts (15). Globally, ANC has proven to be an effective intervention for reducing maternal and infant mortality rates, aligning with Sustainable Development Goal (SDG) 3.1, which aims to reduce maternal mortality to below 70 per 100,000 live births by 2030 (16, 17).

In Senegal, ANC utilization has improved over recent years, thanks to government and organizational efforts to increase access to maternal health services, particularly in rural areas. Policies such as the Maternal Mortality Prevention Program (1990) and the Health Policy (1995) prioritize maternal and reproductive health, aligning with international goals to reduce maternal and neonatal mortality (18). However, barriers to adequate ANC remain, particularly due to the out-of-pocket costs for essential services like diagnostic tests and medications, which limit access for many women in low-income settings (19).

Furthermore, while nearly all pregnant women in Senegal attend at least one ANC visit, only about half complete the minimum recommended four visits. This highlights a quality gap in ANC, with counseling and education often insufficient or entirely lacking (20). To improve maternal and neonatal outcomes, the WHO now advises increasing ANC contacts to at least eight, beginning in the first trimester and continuing throughout the pregnancy (2, 21). Yet, financial difficulties, limited transportation, and sociocultural factors still hinder full ANC attendance, particularly in underserved regions (22–24).

This study analyzes 2023 Demographic and Health Survey (DHS) data to identify factors influencing ANC attendance in Senegal, with emphasis on socioeconomic and regional disparities. Through multilevel Poisson regression analysis, it aims to reveal barriers to ANC utilization, offering insights for policy and resource allocation to improve maternal health and support global health goals.

## Methods

### Study area, data source, and study period

Senegal is located in West Africa, bordered by the Atlantic Ocean to the west and sharing land borders with Mauritania, Mali, Guinea, Guinea-Bissau, and surrounding The Gambia. With a population of over 17 million people as of 2023, Senegal is classified as a low- to middle-income country, with considerable socioeconomic disparities and a predominantly young population. The GDP per capita was reported at approximately $1,641 USD in 2023, reflecting gradual economic growth but with many citizens still living under the poverty line (25).

Administratively, Senegal is divided into 14 regions, each with distinct geographic, cultural, and economic characteristics. These regions include Dakar, Diourbel, Fatick, Kaffrine, Kaolack, Kédougou, Kolda, Louga, Matam, Saint-Louis, Sédhiou, Tambacounda, Thiès, and Ziguinchor. The capital, Dakar, located on the Cape Verde Peninsula, is the country's economic and political hub and exhibits high levels of urbanization. In contrast, other regions, such as Kolda and Sédhiou, are more rural and agrarian, with less developed infrastructure and access to healthcare services (26).

This study utilizes secondary data from the Senegal Demographic and Health Survey 2023. Conducted every five years, the DHS is designed to provide updated health indicators across the country, particularly in the areas of maternal and child health, family planning, vaccination, and healthcare utilization. Data collection for the 2023 survey was a collaborative effort led by the Agence Nationale de la Statistique et de la Démographie (ANSD) with technical assistance from USAID's DHS Program (27).

### Sampling and study population

The 2023 Senegal DHS used a stratified two-stage sampling design. In the first stage, 400 enumeration areas were selected independently for urban and rural strata, comprising 186 urban and 214 rural areas. In the second stage, households within these areas were systematically chosen from household listings.

Several datasets were available, including men's (MR), women's (IR), children's (KR), births (BR), and household (HR) records. For this study, the analysis focused on children's data (KR file), capturing a sample of 4,853 women aged 15 to 49.

The final analysis included women aged 15–49 who reported at least one pregnancy within five years prior to the survey. Cases with incomplete data on ANC attendance, socioeconomic factors, or key variables were excluded, yielding a weighted sample of 4,543 women. This sample size supports robust analysis of factors affecting ANC visits across individual and community levels.

### Study variables

The study's dependent variable is antenatal care attendance, defined as the number of ANC visits each participant attended during her most recent pregnancy. Originally recorded as a range from 0 to 18 visits, ANC visits were recoded to categorize responses as follows: 0 up to 7 visits (i.e., 1, 2, 3 … 7), and ≥8 visits.

The independent variables are categorized into socio-demographic, family and reproductive, and community and health access factors. Socio-demographic factors include age (categorized into age groups: 15–24, 25–34, 35–44, and over 44), women's education level (no education, primary, secondary, or higher), maternal working status (employed or not), husband's education level (no education, primary, secondary and above, or unknown), wealth index (poor, middle, or rich), and household media exposure (access or no access). Family and reproductive factors comprise pregnancy planning status (planned or unplanned), the number of children (0, 1–2, 3–4, or 5 or more), history of terminated pregnancies (yes or no), and autonomy in healthcare decision-making (yes or no). Finally, community and health access factors include distance to a health facility (perceived as a barrier or not), residence type (urban or rural), and region (administrative regions such as Dakar, Ziguinchor, Diourbel, Saint-Louis, among others). These independent variables provide a comprehensive view of both individual and community-level factors potentially influencing ANC utilization in Senegal.

### Data management

This study utilized data from the Demographic and Health Surveys program, which can be accessed at https://www.dhsprogram.com, focusing on outcome and independent variables from the Children's Recode (KR) dataset. Data extraction, coding, and analysis were carried out using Stata version 17, incorporating weighted data (v005) to ensure representativeness at the national level. To enhance accuracy, variables with over 5% missing values or those deemed irrelevant were excluded during data cleaning and preparation.

### Method of data analysis

Researchers often face theoretical challenges when analyzing count variables, which represent the frequency of event occurrences within a given timeframe (e.g., 0, 1, 2, etc.). As count variables can't be negative, they are limited to non-negative integers, including zero (28).

The Poisson regression model is a primary tool for analyzing count data, representing count outcomes as functions of one or more independent variables. This model assumes that observations are independent over time and that the mean and variance of the dependent variable are equal.

Goodness-of-fit tests confirmed the model's adequacy, with a deviance statistic of 3207.311 and a Pearson statistic of 2774.969, both showing *p*-values of 1.000, which indicates a strong fit. The data's mean was 4.24, and the variance was 3.96, resulting in a mean-to-variance ratio of approximately 1.07. This near equivalence supports the equi-dispersion assumption, justifying the use of the Poisson model. Given these results, we applied the Poisson model to interpret predictor effects on ANC visits, while monitoring for any influential observations or residual patterns (29).

The analysis included four model stages. First, a null model (without exposure variables) was used to assess random effects and the feasibility of multi-level modeling. The second model (Model II) introduced individual-level variables to assess their effects on ANC visits. The third model (Model III) added community-level variables, examining the impact of factors such as healthcare access and residence type on ANC visits. The final model (Model IV) combined both individual and community-level factors to evaluate their combined influence on ANC utilization. The multilevel Poisson regression equation used is (30, 31):

log(λit)=β0+β1Xit+β2Zi+ui+ϵit

Where: *λit*, represents the expected number of ANC visits for individual *i* in community *t*, X*it,* denotes the fixed effects of individual-level covariates (such as age, education, and employment status), and Z*i* reflects the random effects of community-level covariates (such as region and access to healthcare). Additionally, ui is the random intercept for community *i*, which accounts for unobserved heterogeneity between communities, while ϵit represents the residual error term for individual *i* in community *t*.

Fixed effects were used to estimate associations between covariates and ANC visits, with variables having *p*-values ≤ 0.20 included in the final model. The adjusted incident rate ratio (AIRR) was presented as the primary measure, with significance set at *p* < 0.05. Random effects evaluated barriers across communities using metrics like the intra-class correlation coefficient (ICC), median incident rate ratio (MIRR), and proportional change in variance (PCV) (32). The variance inflation factor (VIF), with a mean of 4.61, confirmed low multicollinearity. Finally, the model comparison selected the model with the lowest deviance as the best fit.

### Operational definitions

The number of antenatal care visits indicates how many times women, who gave birth in the five years leading up to the survey, received prenatal care for their most recent birth (2).

Autonomy in healthcare decision-making refers to the ability of individuals to make informed choices about their own health and medical care without undue influence or coercion. It emphasizes the importance of personal agency, allowing individuals to express their preferences and take an active role in decisions affecting their health and treatment options (33).

## Results

In a weighted sample of 4,543 pregnant women, the median and mode number of antenatal care visits during pregnancy were four among those who had given birth in the last five years. Overall, 97.63% of women attended at least one ANC visit (95% CI: 0.97, 0.98), and 74.86% received four or more visits (95% CI: 0.72, 0.75) in Senegal. However, only 7.69% of women completed eight or more ANC visits (Table 1).

**Table 1:** Number of women experiencing antenatal care in Senegal, SDHS 2023.

Number of ANC visit	Weighted frequency (n)	Percent (%)
0	95.83	2.11
1	92.15	2.03
2	201.18	4.43
3	752.71	16.57
4	1,599.82	35.21
5	711.17	15.65
6	490.08	10.79
7	250.83	5.52
≥8	349.25	7.69
Mean	4.24	
SD	1.99	
Variance	3.96	

The respondents' mean age was 29.07 years (SD ± 6.91), with most women aged 25–34 years (44.83%), followed by those 15–24 years (30.43%). Women aged 35–44 accounted for 23.48%, while only 1.26% were over 44. In terms of education, over half (54.84%) had no formal education, while 25.66% had secondary education or higher. Among their husbands, 60.67% had no education, with 17.09% reaching secondary level or above.

Regarding wealth status, 46.55% of the women were in the poor category, 34.03% in the rich, and the rest in the middle-income group. Media access was high, with 87.46% of households having access, while 12.54% lacked it.

Most women had 1–2 children (44.91%), while 30.73% had 3–4 children, and 23.66% had five or more. Only 0.70% were childless. Additionally, 17.86% had experienced a pregnancy loss. For healthcare decision-making, 67.94% of women lacked autonomy, with only 32.06% able to make independent healthcare choices.

A majority (58.15%) reported that distance to healthcare facilities was not a barrier. Most women lived in rural areas (62.11%), while 37.89% resided in urban settings. Regional representation was highest in Dakar (18.32%), followed by Thiès (14.07%) and Diourbel (11.27%), with the lowest in Kédougou (1.42%) and Sédhiou (3.66%) (Table 2).

**Table 2:** Socio-demographic characteristics of women who gave birth within the last five years preceding the survey in Senegal, SDHS 2023 (*N* = 4,543).

Variables	Category	Weighted (n)	Percent (%)
Socio-demographic factors			
Age	Mean ± SD	29.07 ± 6.91	
	15–24	1,382.43	30.43
	25–34	2,036.47	44.83
	35–44	1,066.80	23.48
	>44	57.32	1.26
Women education	No education	2,491.29	54.84
	Primary	885.98	19.50
	Secondary	1,165.75	25.66
	Higher	176.05	3.88
Maternal working status	No	3,065.10	67.47
	Yes	1,477.92	32.53
Husband's education	No education	2,756.06	60.67
	Primary	543.43	11.96
	Secondary and above	776.36	17.09
	Don't know	467.17	10.28
Wealth Index	Poor	2,114.93	46.55
	Middle	882.18	19.42
	Rich	1,545.91	34.03
HH Media Exposure	No	569.47	12.54
	Yes	3,973.55	87.46
Family and reproductive factors			
Pregnancy	Planned	3,756.90	82.70
	Unplanned	786.13	17.30
Number of Children	0	31.76	0.70
	1–2	2,040.13	44.91
	3–4	1,396.16	30.73
	≥5	1,074.97	23.66
History of Terminated Pregnancies	No	3,731.55	82.14
	Yes	811.48	17.86
Autonomy to decision of health care	No	3,086.71	67.94
	Yes	1,456.31	32.06
Community and health access factors			
Distance to HF	Not big problem	2,641.80	58.15
	Big problem	1,901.22	41.85
Residence	Urban	1,721.40	37.89
	Rural	2,821.62	62.11
Regions	Dakar	832.41	18.32
	Ziguinchor	123.90	2.73
	Diourbel	512.01	11.27
	Saint-Louis	347.57	7.65
	Tambacounda	229.73	5.06
	Kaolack	379.9	8.36
	Thiès	639.24	14.07
	Louga	253.54	5.58
	Fatick	298.12	6.56
	Kolda	250.15	5.51
	Matam	165.25	3.64
	Kaffrine	280.53	6.17
	Kédougou	64.32	1.42
	Sédhiou	166.35	3.66

### Multilevel poisson regression analysis

#### Random effect, fixed effect and model fitness

Analyzing both fixed and random effects ensures comprehensive insights into ANC utilization. Fixed effects identify individual predictors, while random effects uncover contextual variations across clusters. Together, they integrate personal and structural influences, providing robust conclusions about factors affecting ANC visits at both individual and broader contextual levels.

Following an assessment of multiple count models, the mixed-effects Poisson regression model was selected as the best fit due to the sample's characteristics: the mean number of antenatal care visits (4.24) slightly exceeded the variance (3.96), and the Likelihood Ratio (LR) test yielded a highly significant *p*-value (<0.0001). Additionally, we evaluated for excess zeros to determine if zero-inflated models might be more appropriate, though none proved necessary.

To assess clustering effects, we calculated several measures of variability. The intra-class correlation coefficient indicated that only 0.94% of the total variability in ANC visits was due to cluster effects (Table 3). While this small cluster effect often suggests that a standard Poisson regression model could be enough (with a log-likelihood ratio of −9452.41), we conducted further analysis to ensure robustness (34). A likelihood ratio test comparing the multilevel model to the standard model produced a chi-squared statistic with a *p*-value of <0.001, supporting the multilevel model by rejecting the null hypothesis of no between-cluster variability in ANC visit rates.

**Table 3:** Parameters and model fitness statistics for multilevel poisson regression analysis.

Parameters	Null model	Model 2	Model 3	Model 4
Cluster level variance (SE)	0.0269855 (0.0036254)	0.0153675 (0.0027139)	0.0122523 (0.0023937)	0.0090926 (0.0021536)
ICC	0.94%	0.48%	0.37%	0.29%
MIRR	1.182 (95% CI = 1.02, 1.04)	1.124 (95% CI = 1.10, 1.15)	1.11 (95% CI = 1.09, 1.14)	1.0947 (95% CI = 1.07, 1.12)
PCV	reference	42.85%	54.66%	66.54%
LLR	−9491.35	−9411.77	−9421.01	−9370.67
Deviance	18982.701	18823.542	18842.03	18741.336

SE, standard error; ICC, intraclass correlation; MIRR, median incident rate ratio; PCV, proportional change in variance; LLR, loglikelihood ratio.

The Median Incident Rate Ratio (MIRR) provided additional insights into cluster-level variation, measuring the median relative difference in ANC visit rates between high- and low-visit clusters. A significant MIRR of 1.18 (95% CI: 1.02–1.04) affirmed that the multilevel model captured meaningful variation beyond the standard model (35).

We further evaluated the Percent Change in Variance (PCV) to quantify the variation explained by individual- and cluster-level factors. The deviance test, with values progressively decreasing from the null model to Model IV, confirmed Model IV as the best fit. The final model explained approximately 66.54% of the total variability in ANC visits, underscoring its effectiveness (Table 3).

In the final model, several factors were significantly associated with the frequency of ANC visits in Senegal, with wealth status, household media exposure, husband's education, and regional location playing key roles (Table 4).

**Table 4:** Multilevel poisson regression on the number of antenatal care visits among women who gave birth within the last five years preceding the survey in Senegal, SDHS 2023.

Variables	Category	Model II (AIRR (95% CI)	Model III (AIRR (95% CI)	Model IV (AIRR (95% CI)
Maternal age	15–24	1.00		1.00
	25–34	1.00 (0.97, 1.04)		1.00 (0.96,1.04)
	35–44	1.00 (0.96, 1.06)		1.01 (0.96,1.06)
	>44	0.96 (0.84, 1.10)		0.96 (0.84,1.10)
Maternal education	No education	1.00		1.00
	Primary	1.01 (0.97, 1.05)		1.01 (0.97, 1.05)
	Secondary	1.03 (0.99, 1.07)		1.03 (0.98, 1.07)
	Higher	1.10 (1.01, 1.21)*		1.10 (1.01, 1.21)*
Wealth status	Poor	1.00		1.00
	Middle	1.08 (1.04, 1.13)**		1.06 (1.02, 1.11)*
	Rich	1.13 (1.08, 1.18)**		1.08 (1.03, 1.14)*
Maternal working status	No	1.00		1.00
	Yes	0.99 (0.97, 1.03)		0.99 (0.97, 1.03)
Pregnancy	Planned	1.00		1.00
	Unplanned	0.97 (0.93, 1.01)		0.97 (0.93, 1.01)
Autonomy to the decision of healthcare	No	1.00		1.00
	Yes	0.99 (0.96, 1.02)		0.99 (0.96, 1.02)
History of terminated pregnancy	No	1.00		1.00
	Yes	1.01 (0.97, 1.05)		1.02 (0.98, 1.05)
Husband Education	No education	1.00		1.00
	Primary	1.06 (1.01, 1.11)*		1.06 (1.01, 1.11)*
	Secondary	1.07 (1.02, 1.13)*		1.08 (1.02, 1.13)*
	Higher	1.10 (1.03, 1.17)*		1.09 (1.02, 1.16)*
	Don’t know	1.02 (0.97, 1.07)		1.03 (0.97, 1.08)
HH media exposure	No	1.00		1.00
	Yes	1.11 (1.06, 1.16)**		1.10 (1.05, 1.15)**
Number of children	0	1.00		1.00
	1–2	1.00 (0.85, 1.18)		1.00 (0.85, 1.18)
	3–4	0.96 (0.82, 1.13)		0.97 (0.82, 1.14)
	≥5	0.92 (0.78, 1.07)		0.93 (0.79, 1.09)
Distance to health facility	Not big problem		1.00	1.00
	Big problem		1.00 (0.97, 1.03)	0.98 (0.95, 1.02)
Residence	Urban		1.00	1.00
	Rural		0.89 (0.86, 0.93)	0.96 (0.92, 1.01)
Regions	Dakar		1.00	1.00
	Ziguinchor		0.92 (0.83, 1.02)	0.95 (0.85, 1.05)
	Diourbel		0.93 (0.85, 1.02)	0.94 (0.86, 1.03)
	Saint-Louis		0.91 (0.83, 1.00)	0.94 (0.86, 1.03)
	Tambacounda		0.83 (0.75, 0.91)**	0.90 (0.82, 0.99)*
	Kaolack		0.96 (0.88, 1.06)	0.99 (0.91, 1.10)
	Thiès		0.79 (0.72, 0.87)**	0.80 (0.73, 0.87)**
	Louga		1.00 (0.91, 1.10)	1.03 (0.94, 1.12)
	Fatick		0.87 (0.79, 0.96)*	0.90 (0.83, 0.99)*
	Kolda		0.92 (0.84, 1.01)	0.97 (0.88, 1.06)
	Matam		0.77 (0.7, 0.85)**	0.83 (0.75, 0.92)**
	Kaffrine		0.82 (0.75, 0.90)**	0.87 (0.80, 0.97)*
	Kédougou		0.78 (0.71, 0.86)**	0.83 (0.75, 0.91)**
	Sédhiou		0.75 (0.68, 0.82)**	0.80 (0.73, 0.88)**

* *p*-value <0.05.

** *p*-value < 0.001.

AIRR, adjusted incidence rate ratio.

Women from middle- and high-income households attended significantly more ANC visits compared to those from poorer households. Specifically, women in middle-income households had a 6% higher rate of ANC visits (AIRR: 1.06, 95% CI: 1.02–1.11), while women in high-income households had an 8% higher rate of visits (AIRR: 1.08, 95% CI: 1.03–1.14). Additionally, women in households with media exposure had 11% more ANC visits than those without media access (AIRR: 1.11, 95% CI: 1.06–1.15).

Educational attainment also influenced ANC attendance. Women with higher education had 10% more ANC visits compared to those with no formal education. The husband's education was also positively associated with ANC attendance: women whose husbands had a primary education attended 6% more ANC visits (AIRR: 1.07, 95% CI: 1.01–1.11), those whose husbands completed secondary education attended 8% more visits (AIRR: 1.08, 95% CI: 1.02–1.13), and women with husbands who attained higher education had 9% more visits (AIRR: 1.09, 95% CI: 1.02–1.16), all compared to women with husbands who had no formal education.

Regional disparities were observed. Women in the Thiès region were 20% less likely to attend ANC visits (AIRR: 0.80, 95% CI: 0.73–0.87), and those from Matam (AIRR: 0.83, 95% CI: 0.75–0.92), Kédougou (AIRR: 0.83, 95% CI: 0.75–0.91), and Sédhiou (AIRR: 0.80, 95% CI: 0.73–0.88) also had significantly fewer visits. Additionally, women from Tambacounda (AIRR: 0.90, 95% CI: 0.82–0.99) and Fatick (AIRR: 0.90, 95% CI: 0.83–0.99) showed a lower likelihood of attending ANC visits compared to those residing in Dakar.

## Discussions

Antenatal care is a critical indicator of maternal healthcare, essential for reducing maternal and child mortality and supporting global health objectives (36). In this study, 97.63% of Senegalese women attended at least one ANC visit (95% CI: 0.97, 0.98), with 72.39% achieving the minimum four visits (95% CI: 0.71, 0.74). However, only 7.69% met the WHO-recommended eight visits, a rate higher than Gabon (3.66%) but lower than Nigeria (19.17%) and consistent with sub-Saharan Africa's average (8.9%) (37, 38).

Since 2016, WHO guidelines recommend eight ANC contacts to reduce perinatal mortality and improve care experiences (39). This analysis, using multilevel Poisson regression and recent national data, highlights ANC utilization gaps in Senegal, influenced by factors such as maternal and partner education, wealth index, media exposure, and geographic region.

This study shows that women with higher education were 10% more likely to attend ANC visits than those without formal education, emphasizing the positive role of education in maternal healthcare utilization. This association between education and ANC attendance is well-documented across various countries, including Senegal (40), Ghana (41), Cameroon (42), Zambia (43), Uganda (44), Ethiopia (45), Bangladesh (46), Nepal (47), East Africa (48), and Sub-Saharan Africa (49).

Increased educational attainment in low- and middle-income countries (LMICs) often correlates with better health outcomes and healthcare access. Educated women tend to have greater autonomy, health literacy, and decision-making power, leading to higher ANC attendance through improved awareness of prenatal care, as well as overcoming logistical and financial barriers (36, 43, 47, 48).

Additionally, this study found that a husband's education level also influences women's ANC attendance. Women whose husbands had primary education were 6% more likely to attend ANC visits, with this likelihood rising to 8% and 9% when husbands had secondary or higher education. This underscores the supportive role that educated husbands can play in maternal healthcare, as seen in studies from Ethiopia (50), Nepal (51, 52), Indonesia (53), Pakistan (50), East Africa (54), and Sub-Saharan Africa (55, 56).

Educated husbands are generally more aware of the benefits of maternal healthcare, and they provide both financial and logistical support. They often prioritize healthcare spending and encourage their wives to seek timely care, particularly in patriarchal societies where men frequently make household healthcare decisions (57–59).

Women from middle-income households are 6% more likely, and those from high-income households 8% more likely, to attend ANC visits compared to women from low-income households, suggesting that financial resources in these households mitigate barriers like transportation costs, clinic fees, and time off work, promoting consistent ANC attendance (60). Studies from Angola (61), Ethiopia (36, 49), Guinea (62), Ghana (63), Nigeria (3), Sub-Saharan Africa (49), Nepal (64) reinforce this association, highlighting that economic stability enhances ANC use by improving health information access, covering expenses, and fostering autonomy in health decisions.

In Senegal, however, the income-related increase in ANC attendance is relatively modest (6% for middle-income and 8% for high-income households), likely due to additional factors facilitating ANC access across income groups. Senegal's healthcare infrastructure, with extensive facilities, shorter travel distances, and affordable or free transport, fosters more equitable access (65, 66). Although ANC services are not entirely free, essential services like malaria prophylaxis, anti-tetanus vaccinations, and HIV testing are often provided at no cost (19), reducing financial obstacles for low-income households.

Media exposure also significantly affects health-seeking behaviors, as women with regular media access are 10% more likely to attend ANC visits than those with limited exposure. This trend, observed in Nigeria (67), Uganda (68), Sub-Saharan (49), India (69), Nepal (70), and Bangladesh (71) demonstrates how media access increases health literacy and engagement with maternal healthcare by providing information on prenatal care benefits, services, and local initiatives (72). Additionally, media exposure fosters a supportive environment for ANC, especially in traditional societies, where family support for healthcare is crucial (73).

Regional disparities within Senegal further illustrate these trends. Compared to Dakar, regions like Tambacounda (IRR: 0.90, 95% CI: 0.82–0.99), Thiès (0.80, 95% CI: 0.73–0.87), Fatick (0.90, 95% CI: 0.83–0.99), Matam (0.83, 95% CI: 0.75–0.92), Kaffrine (0.87, 95% CI: 0.80–0.97), Kédougou (0.83, 95% CI: 0.75–0.91), and Sédhiou (0.80, 95% CI: 0.73–0.88) exhibit lower adjusted incidence rate ratios (IRRs) for ANC visits, likely due to factors beyond income. Dakar, with 19% of hospital users, benefits from 38% of the national hospital budget, creating a resource concentration that leaves regions like Fatick, with only 2% of healthcare resources, underserved despite high primary healthcare demand (74).

Urban-rural disparities in access further affect ANC attendance. Dakar has a robust transport network, whereas remote areas like Sédhiou face limited public transport options, increasing indirect costs such as travel expenses and restricting regular ANC access (75). Rural regions also struggle with healthcare worker shortages; Dakar's healthcare worker density is significantly higher than in regions like Kaffrine and Kédougou, where retaining trained staff is a challenge (65). Together, these disparities in infrastructure, economic resources, and workforce availability contribute to lower ANC attendance in these underserved areas relative to Dakar (60).

This study utilizes nationally representative DHS data, analyzed with a multilevel Poisson regression model to account for count-based ANC visits and hierarchical data structures, enhancing methodological rigor. However, its cross-sectional design limits causal inference, and self-reported measures may introduce bias. While perceived distance to healthcare facilities (measured as a self-reported barrier) was not statistically significant, incorporating geospatial (GIS) analysis in future research could provide deeper insights into the role of geographic distance.

## Conclusions

This study underscores persistent inequities in antenatal care utilization in Senegal, particularly among rural women, those with limited education, and low-income households. While national progress has been made in expanding ANC access, structural barriers such as uneven healthcare resource distribution, financial constraints, and gaps in health literacy continue to hinder equitable maternal care. Addressing these disparities is essential to advancing maternal and child health outcomes and achieving Senegal's commitments to national and global health equity goals.

To reduce these disparities, targeted policy interventions are necessary. Expanding community-based maternal health education programs, including those for both women and men, can increase ANC attendance. Strengthening healthcare infrastructure and distributing healthcare workers more evenly, especially in underserved regions like Tambacounda and Kaffrine, is crucial. Additionally, providing financial support such as transportation subsidies will help improve access for low-income households. Collaborating with local media to promote maternal health information can enhance health literacy, particularly in rural areas.

Improving transportation infrastructure will further reduce costs and improve overall access to care. Moreover, incorporating spatial analysis into future research will provide insights into the impact of physical proximity on ANC access, enabling the development of targeted interventions, such as mobile clinics in remote regions.

## Data Availability

The original contributions presented in the study are included in the article/Supplementary Material, further inquiries can be directed to the corresponding author/s.
